# A major depression prognosis calculator based on episode duration

**DOI:** 10.1186/1745-0179-2-13

**Published:** 2006-06-14

**Authors:** Scott B Patten

**Affiliations:** 1 Professor, Department of Community Health Sciences & Department of Psychiatry. University of Calgary, Calgary, Alberta, Canada

## Abstract

**Background:**

Epidemiological data have shown that the probability of recovery from an episode declines with increasing episode duration, such that the duration of an episode may be an important factor in determining whether treatment is required. The objective of this study is to incorporate episode duration data into a calculator predicting the probability of recovery during a specified interval of time.

**Methods:**

Data from two Canadian epidemiological studies were used, both studies were components of a program undertaken by the Canadian national statistical agency. One component was a cross-sectional psychiatric epidemiological survey (n = 36,984) and the other was a longitudinal study (n = 17,262).

**Results:**

A Weibull distribution provided a good description of episode durations reported by subjects with major depression in the cross-sectional survey. This distribution was used to develop a discrete event simulation model for episode duration calibrated using the longitudinal data. The resulting estimates were then incorporated into a predictive calculator. During the early weeks of an episode, recovery probabilities are high. The model predicts that approximately 20% will recover in the first week after diagnostic criteria for major depression are met. However, after six months of illness, recovery during a subsequent week is less than 1%.

**Conclusion:**

The duration of an episode is relevant to the probability of recovery. This epidemiological feature of depressive disorders can inform prognostic judgments. Watchful waiting may be an appropriate strategy for mild episodes of recent onset, but the risks and benefits of this strategy must be assessed in relation to time since onset of the episode.

## Background

Clinical practice guidelines (CPGs) provide important mechanisms for integrating scientific evidence with clinical care. The process of developing guidelines, however, depends largely on data from randomized controlled trials, and the resulting recommendations are not always generalizable to real-world populations. Most CPGs in North America regard fulfillment of DSM-IV criteria for major depression as a *de facto *indication for pharmacological or non-pharmacological treatment e.g. [1], whereas the UK National Institute for Health and Clinical Excellence (NICE) guidelines indicate that mild episodes may not require treatment with antidepressants [2].

These apparent inconsistencies relate to a broader debate within psychiatric epidemiology concerning the clinical significance of depressive episodes identified in community studies. Attention has been drawn to this issue by Narrow et al. [3] who found that including items relating to "clinical significance" in diagnostic algorithms applied to data from structured diagnostic interviews can substantially alter prevalence estimates. More recently, Brugha et al. have highlighted a similar issue by noting poor agreement between results from a fully structured lay administered diagnostic interview and a semi-structured interview administered by clinicians [4,5].

Epidemiological data about the prognosis of episodes can help to inform clinical decisions. Much of the research on determinants of episode duration has been conducted in clinical samples [6,7], but community studies have also been published. The Netherlands Mental Health Survey and Incidence Study (NEMESIS) reported that physical illness, lack of social support, severity of depression and having had a previous long episode were all associated with increased duration of an index major depressive episode [8]. In an analysis of incident cases in NEMESIS, Spijker et al. found that demographic variables did not predict episode duration [9]. It was noted that about half of such episodes resolved within three months, but that the rate of recovery appeared to slow over time. An association between previous severe recurrences and the duration of more recent episodes was interpreted as possible evidence of a "scar" effect [10]. Another source of episode duration data is the NIMH Collaborative Depression Study [11]. A key result from this study was that the probability of recovery declined with increasing episode duration [12]. The same finding was suggested by models fit to data from the Canadian National Population Health Survey (NPHS) [13-15]. The modeling approach used in the latter study should give similar results to the approach employed here, which used the Weibull distribution (see below, Methods).

## Methods

The Canadian Community Health Survey, Mental Health and Well-being (CCHS 1.2) was a national survey conducted in 2002. The target population consisted of residents of private dwellings (ie. non-institutionalized or homeless) who were aged 15 years or older in the 10 Canadian provinces. The survey had a sample size of 36,984 and achieved a response rate of 77%. All subjects were administered a Canadian version of the World Mental Health (WMH) Composite International Diagnostic Interview (CIDI) developed for the World Mental Health 2000 project and known as the WMH-CIDI [16]. Detailed methodological information about the CCHS 1.2 has recently been reported [17] and additional information is available on-line, including an electronic copy of the WMH-CIDI version used [18].

As noted above, available evidence suggests that the probability of recovery from a major depressive episode declines with increasing episode duration. In engineering applications, a situation emerges which is analogous to this. Engineers attempting to model the rate of failure of machinery often observe that the rate of failure is not constant over time, but rather increases as the machine gets older. The Weibull distribution can be used to model this situation. In the current study, application of the Weibull distribution to describe major depressive episode duration was explored. Subsequently, a simulation model was developed and calibrated using a longitudinal data source. A prognosis "calculator" depicting the relationship between episode duration and prognosis was also made.

The version of the CIDI used in the CCHS 1.2 includes inquiries about the length of first episodes for subjects reporting multiple episodes of major depression. These data were used to examine the usefulness of the Weibull model for describing the duration of episodes. STATA 8.0 [19] was used to fit a Weibull model to the data using a least squares non-linear modeling procedure (the STATA 'nl' command) for Weibull and exponential models, the latter representing a situation where there is a constant rate of recovery.

The National Population Health Survey (NPHS) is a longitudinal study that began in 1994 with the selection of a representative sample of 17,262 from the Canadian general population. Subjects have been re-interviewed every two years since then, in 1996, 1998, 2000, 2002 and 2004, although data from the 2004 interview have not been released. In this paper, the intervals between these interviews are referred to as "cycles" such that 1994 to 1996 is Cycle 1, 1996 to 1998 is Cycle 2 and so on. The NPHS interview included the CIDI Short Form for major depression (CIDI-SFMD) [20], which is a brief predictive interview that assesses 12-month period prevalence of major depression. The positive predictive value of the CIDI-SFMD for CIDI-defined major depressive episode is probably between 75% and 90% [20,21]. Using the NPHS, it is possible to estimate an approximation of annual incidence: the proportion of the cohort that were CIDI-SFMD negative at the beginning of a cycle (e.g. 1994), who were positive at their next interview at the end of the cycle (in this case 1996). The NPHS included an item for those positive on the CIDI-SFMD, asking about weeks depressed in the past year. This variable is related to, but not exactly equivalent to, episode duration.

Discrete event simulation modeling used the software Arena [22], which is one of several commercially available programs that provide a graphical interface for developing simulation models in the SIMAN language. The simulation model was set up to accommodate the previously mentioned idiosyncrasies of the NPHS study design: (a) the assessment of annual (past year) major depression prevalence when the interviews occurred two years apart, and (b) the measurement of weeks depressed in the past year rather than episode duration. An annual incidence rate and the two parameters that define a Weibull distribution (a "scale" and "shape" parameter) were considered inputs, or "controls" in the model. Simulated weeks depressed in past year was one output, as was the simulated proportion of subjects without major depression at the start of the cycle who had two weeks or more of depression during the final 365 days of the 730 day simulation cycle. This proportion is described using the term "approximate incidence proportion" in the remainder of the manuscript.

Another software program, called OptQuest [23], was used for the simulation analyses to identify values for the three inputs (annual incidence, scale and shape) that would lead to simulated outputs most closely approximating the NPHS results. OptQuest works by running replicated simulations using different values for input variables and finding those that minimize or maximize specified outputs. To assist with model calibration, two sum of squared difference variables were created: (a) the sum of squared differences between the observed and simulated approximate incidence proportions and (b) the sum of squared differences between the simulated and observed frequencies of weeks depressed in the past year. OptQuest was used to identify input values that would minimize these two variables, thereby finding a set of inputs that would, according to the model, lead to the observed NPHS data.

Incidence, and the Weibull parameters were calculated for each of the four available cycles, and quantile-quantile plots of simulated versus observed data were used to assess the adequacy of the simulations. The cumulative distribution for major depression episode duration, according to the parameter estimates from the model, were placed into an Excel^® ^spreadsheet projecting the probability of recovery as a function of episode duration. The spreadsheet contains four macros, so that by clicking one of four buttons the user can substitute Weibull estimates derived from any of the four cycles in the calculation of the cumulative recovery probabilities. The spreadsheet can be downloaded through the Additional File 1 link associated with this paper.

## Results

In the CCHS 1.2, there were 4,713 subjects with lifetime major depression, representing a weighted lifetime prevalence of 12.2%. Of these subjects, 1944 reported an episode of major depression in the 12 months preceding their interview (weighted 12-month prevalence 4.8%). Episode duration data were collected using items that asked about the length of the first episode. This question was asked to 2905 subjects who reported having at least two lifetime episodes. There were 264 subjects who did not respond to this item, such that complete data was collected from 2641 (90.9%) of relevant subjects.

Table 1 presents duration data for first episodes, as recorded in the data file. For the modeling, it was necessary to convert the episode durations into common units, and weeks were chosen for this purpose. Ranges were used to record the duration of the two longest categories in the data file and in the conversion of these categories to weeks used the lower bound of the range. Subjects reporting 2 to 4 year episode durations were coded as 104 weeks, and those reporting five or more were coded as 260 weeks. An alternative strategy would have been to use the midpoint of the range, but this was not possible as the upper range did not have an upper bound. Many of the episodes were short lived, with 16% of episodes having a reported duration of 2 weeks, which is the minimum duration required by the DSM-IV [24] and ICD-10 [25] criteria. Nevertheless, 13.7% of the subjects reported that their first episode lasted 5 years or longer. With conversion of units from months and years to weeks, the median duration of first episodes was 17.3 weeks, or approximately four months. The overall pattern resembles that seen in other community studies (see review, [26]).

**Table 1:** Reported lengths of first episodes, respondents reporting 2 or more lifetime episodes, Canadian Community Health Survey 1.2.

Episode duration (as reported)	Episode duration converted to weeks for non-linear model fitting (weeks)	N (%)	Cumulative (%)
2 weeks	2	459 (16.0)	16.04
3 weeks	3	170 (5.9)	21.98
1 month	4.3	245 (8.6)	30.54
2 months	8.7	285 (10.0)	40.50
3 months	13	230 (8.0)	48.5
4 months	17.3	91 (3.2)	51.71
5 months	21.7	47 (1.6)	53.35
6 months	26	239 (8.4)	61.71
7 months	30.3	20 (0.7)	62.40
8 months	34.7	46 (1.6)	64.01
9 months	39	17 (0.6)	64.61
10 months	43.3	28 (1.0)	65.58
11 months	47.7	10 (0.4)	65.93
1 year	52	240 (8.4	74.32
2 years*	104	343 (12.0)	86.30
5 years*	260	392 (13.7)	--
Total		2,862 (100)	

* Responses were categorized as ranges: 2 – 4 years and 5 or more years. For consistency, the start of the range was used in the modeling, see text for further explanation.

The probability of recovery by time was related to a two parameter Weibull distribution, with a scale parameter 'a' and shape parameter 'b':

Cumulative Probability of Recovery = 1 - exp[-(time/a)^b^]

**Figure 1:**
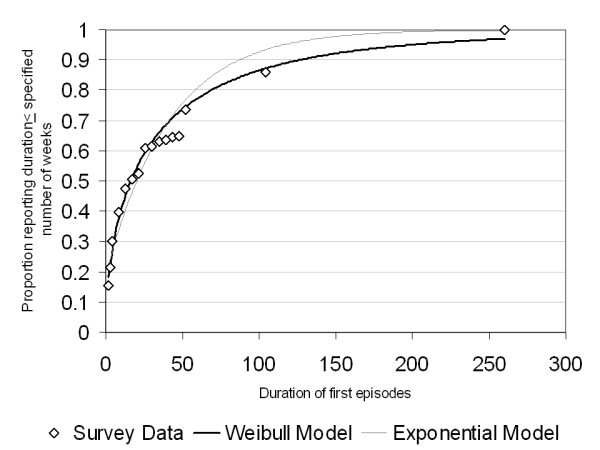
Fitted Values for First Episode Duration, Major Depression Data from the Canadian Community Health Survey 1.2.

If the recovery probability does not decline over time, then b = 1 and the Weibull distribution becomes an exponential distribution. Non-linear modeling found that the best-fitting scale parameter had a value less than one: 0.56. This is consistent with the idea that the recovery probability declines with time. A non-linear model using the exponential distribution (constant rate of recovery over time) tended to underestimate the proportion recovering in early weeks and overestimate it in later weeks (see Figure 1). Equivalent results were obtained using two other methods of relating the cumulative recovery probability to the Weibull distribution: linear regression of log(-log [1 - recovery proportion by time (t)]) against log time, in which case the slope of the regression line is 'b' and the intercept is -b(log(a)). Finally, a generalized linear model for cumulative recovery was fit with log time as the predictor variable, and using a complementary log-log link function in STATA. The fitted values using each approach were nearly identical.

**Figure 2:**
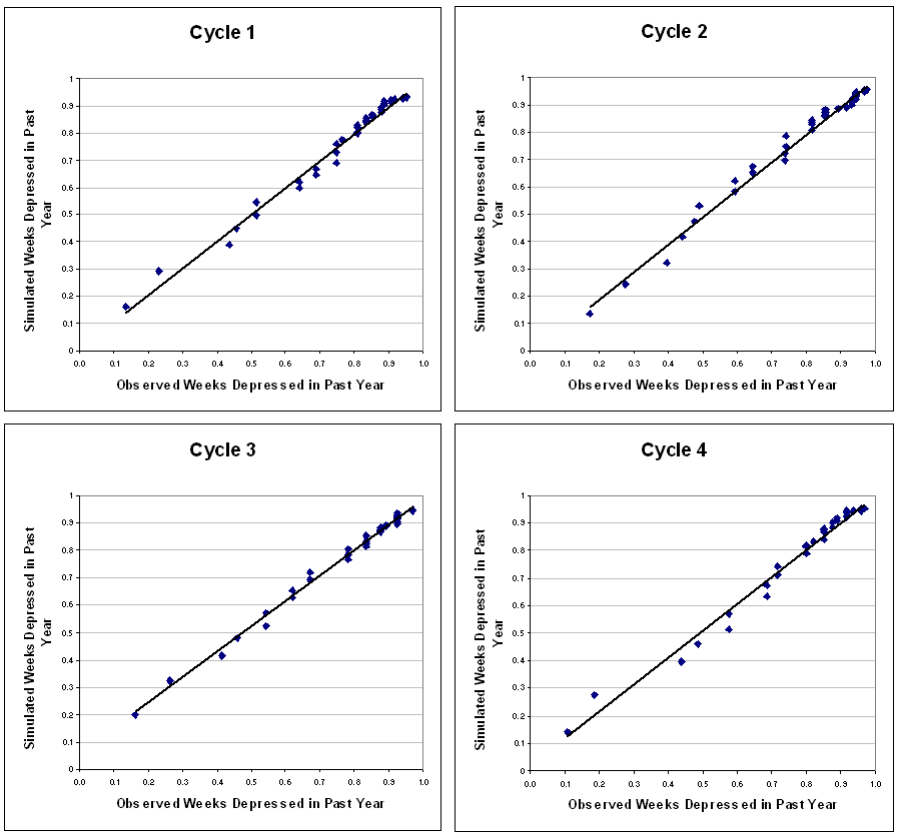
Quantile-Quantile Plots of Observed Versus Simulated Weeks Depressed in Past Year from 4 National Population Health Survey (NPHS) Cycles.

Table 2 presents OptQuest solutions for the approximate incidence, annual incidence, scale and shape values for Cycles 1 through 4 of the NPHS. Figure 2 presents quantile-quantile plots for the simulated versus observed cumulative number of weeks depressed in the past year. Additional File 1 uses the Weibull recovery parameters to calculate prognosis as a function of episode duration.

**Table 2:** Estimated annual incidence and Weibull parameters for major depression

Year	N	Approximate incidence proportion*	Model-Based Estimates			
			
			Approximate Incidence Proportion*	Adjusted Annual Incidence**	Weibull Scale Parameter	Weibull Shape Parameter
1994–96	9370	3.5%	3.5%	2.8%	52.9	0.66
1996–98	9689	3.6%	3.6%	2.8%	52.9	0.66
1998–00	9427	3.8%	3.8%	3.1%	47.4	0.71
2000–02	9244	3.8%	3.8%	3.1%	47.9	0.86

* proportion without an episode in the year preceding the start of the interval (1994, 1996, 1998 or 2000) who have an episode in the year preceding the following interview.

** using the exponential formula (2 year incidence = 1 - e^-one year rate*2^) these annual rates correspond to predicted 2-year incidence ranging from 5.4% to 6.0%.

## Conclusion

The models and data presented here are consistent with the idea that the probability of recovery from major depressive episodes diminishes with increasing episode duration, as suggested by previous studies. Changes that occur over time as episodes unfold may be related to a diminished propensity for recovery. For example, neurotoxicity or a failure of neurogenesis, see reviews [27,28] may lead to decreasing hippocampal cellular reserves as episodes get longer. In turn, this may be related to a diminishing propensity for recovery. Similarly, cognitive, behavioural and social changes that occur during depression may become more entrenched and habitual with increasing episode duration.

In more practical terms, these result are consistent with the idea that a large number of depressive episodes occurring in community populations resolve quickly. Since many episodes last only a few weeks, the results seem consistent with the idea that not all major depressive episodes require treatment, an idea endorsed by the NICE guidelines. This conclusion seems even more evident when one considers that treatment generally takes several weeks to be effective. However, a significant qualification applies to this finding. In these models, "prevalent" cases of depression were excluded at the baseline time point for each cycle. This was done so that the frequency of episodes at the end of a cycle would approximate incidence rather than prevalence. Projections beyond the two year time frame must be considered speculative by virtue of the modeling methods employed. However, the tendency of recovery rates to decline with increasing episode duration implies that over time there is likely to be an accumulation of chronic episodes in the population. As such, whereas new episodes appear often to be of brief duration this does not necessarily mean that episodes detected, for example, during screening in primary care are necessarily brief. These may be prevalent cases that have been undetected and may represent longstanding episodes that have accumulated in the population. This distinction may be an important one clinically. In one sense, the results presented here seem consistent with the idea that "watchful waiting" may be a reasonable strategy for mild depression, but they also imply that decisions about how long to wait should be made with reference not to the time of detection or clinical presentation, but rather with reference to the time of onset of an episode.

The fitted values from NPHS are presented in an electronic attachment to this paper, Additional File 2. The study estimates of incidence and Weibull scale and shape are depicted in the form of a 14 day animation of the NPHS, visually depicting the epidemiology as described by the models. This particular animation incorporates the incidence and duration estimates from the 2000–2002 NPHS cycle. The animation illustrates that even though most new episodes are brief, there is an accumulation of longer episodes in the prevalence pool.

The concept of persistence is central to existing definitions of depressive episodes. The concept of duration is one of the ways in which these definitions attempt to distinguish between episodes that are or are not clinically relevant. The general idea of including persistence in diagnostic definitions receives support from these results in the sense that duration seems strongly related to prognosis, but as the probability of recovery appears to decline as a function of episode duration, the use of any particular time frame, such as the two week reference period used by DSM [29] and ICD-10 [25] seems inadequate. The output of the calculator indicates that an episode that has only lasted a few weeks is likely to be associated with a high rate of recovery in the following few weeks, whereas an episode that has lasted for many weeks has a low probability of recovery in the next few weeks. It may be more meaningful to treat episode duration as a dimensional quality at the time of presentation, incorporating epidemiological data into clinical judgments based on this quantity.

These results also have implications for screening in clinical settings. In primary care, active case-finding is often an element of disease management strategies, e.g. [30-34], see also a review by Katon [35]. These results suggest that early detection by screening may potentially result in identification of a subgroup with a much better prognosis, and perhaps with a sizable likelihood of recovery even if untreated. Screening measures should be carefully organized and monitored so that they do not cause a diversion of resources away from individuals with greater needs. Such a diversion will not necessarily occur, but could occur if screening resulted primarily in the detection of brief and self-limited episodes. According to the calculator, a person with a three week duration of symptoms has an approximately 40% chance of recovery in the next six weeks, whereas a person with a 23 week duration has less than a 5% chance over the same interval. The risks and benefits of depression screening in various clinical populations will depend not only on the basic features that are usually considered: the prevalence of depression in those populations, the sensitivity and specificity of the measures employed, but also on help-seeking behavior and health systems issues. While traditional screening assessments are based on symptom rating scales or subsets of related questions, items about symptom duration may be more critical than has previously been believed.

The very high probability of recovery in the early weeks of a major depressive episode, combined with the necessity for several weeks of treatment before a response is expected, may suggest that many people with short-lived symptoms do not need active treatment. It is possible, as noted above, that the reasons for the declining recovery rate as episodes get longer is a result of secondary effects of the depression itself. This possibility creates an argument for earlier intervention, rather than expectant management. Further research will be needed to resolve these questions.

The calculator presented in this paper helps to illustrate the importance of episode duration on the probability of recovery in the near future. With the development of more sophisticated episode duration models, it should be possible to develop calculators such as this one into more sophisticated decision-support tools. A step in this direction would involve incorporating other predictors of duration into the calculator's algorithms. However, such efforts need to account for the apparent relationship between episode duration and recovery probability and should not be restricted to estimating average frequencies of recovery or mean episode durations. Furthermore, in view of an apparent change in the probability of recovery over time, classical survival analysis models (particularly those requiring a constant hazard function) are probably not adequate for this task.

## Supplementary Material

Additional File 1 This is an Excel^® ^spreadsheet with an embedded macro for calculating the probability of recovery during a specified interval of time using estimates from the models presented in this paper. You may need to alter the security settings in Excel^® ^in order to use the calculator. If the program is set to high security, it may not allow macros to run. To change these settings, click Tools then Options then Macro Security. Click here for file

Additional File 2 This is an "AVI" video file presenting an animation of the model described in the paper. The animation illustrates that even though most new episodes are brief, an accumulation of longer episodes in the prevalence pool is predicted by the model. Click here for file
